# Immunoglobulin binding protein (BiP) forms two types of dimers

**DOI:** 10.1002/pro.70690

**Published:** 2026-07-09

**Authors:** Karina New, Miguel I. A. Lagos‐Espinoza, Nathalie Casanova‐Morales, Roi Asor, John William Young, Zahra Alavi, Christian A. M. Wilson

**Affiliations:** ^1^ Departamento de Bioquímica y Biología Molecular, Facultad de Ciencias Químicas y Farmacéuticas Universidad de Chile Santiago Chile; ^2^ Facultad de Artes Liberales Universidad Adolfo Ibáñez Santiago Chile; ^3^ Physical and Theoretical Chemistry Laboratory, Department of Chemistry University of Oxford Oxford UK; ^4^ The Kavli Institute for Nanoscience Discovery Oxford UK; ^5^ Department of Physics Loyola Marymount University Los Angeles California USA

**Keywords:** chaperone protein, dimerization, endoplasmic reticulum, Hsp70, mass photometry, protein–protein interactions, proteostasis, unfolded protein response

## Abstract

Immunoglobulin binding protein (BiP) is a chaperone protein that plays crucial roles in protein folding and transport by binding unfolded proteins in the substrate binding domain (SBD), an interaction allosterically linked to the nucleotide occupancy of the nucleotide binding domain (NBD). BiP also forms oligomers that influence its activity, particularly in binding polypeptide clients. Using single‐molecule approaches and mechanical and enzymatic activity assays, BiP monomer stability and oligomerization were analyzed both alone and in response to nucleotides and peptides. We find that the formation of BiP dimers exhibits biphasic behavior as a function of BiP concentration, suggesting concentration‐dependent changes in dimer dissociation constants. At low BiP concentrations, dimers were disrupted by peptide substrate and adenosine triphosphate (ATP) but remained unaffected by ADP or Adenosine 5′‐O‐(3‐thio)triphosphate (ATPγS). At high concentrations, dimers are unaffected by peptides, but their assembly is inhibited by ATP and ATPγS to the same degree. These results suggest the formation of two distinct BiP dimers exhibiting unique binding affinities, kinetics, and potentially structures. We propose the existence of a high‐affinity dimer binding site within the SBD of BiP, while the formation of a low‐affinity dimer involves interactions between the lid and NBD of each protomer. Our findings demonstrate the importance of considering single‐molecule characteristics when interpreting bulk studies on protein function and regulation.

## 1 INTRODUCTION

Immunoglobulin binding protein (BiP, also called HSPA5, Grp78, or Kar2) is an ATPase that has been referred to as the master regulator of the endoplasmic reticulum (ER) due to its multiple roles (Alfaro‐Valdés et al., 2018; Quiroga et al., 2022; Wilson et al., 2025). It is essential for post‐translational protein translocation, acts as a chaperone in protein folding, and activates the unfolded protein response (UPR) (Pobre et al., 2019). The concentration of BiP in the ER lumen is in the order of micro to millimolar, being the third most abundant protein in our cells (Bakunts et al., 2017; Ghaemmaghami et al., 2003; Guth et al., 2004; Lai et al., 2010; Zimmermann & Lang, 2020).

BiP is formed of two allosterically associated domains connected by a linker: the substrate binding domain (SBD) that binds unfolded polypeptides, and the nucleotide binding domain (NBD) that binds ATP or ADP (Blond‐Elguindi, 1993; Pobre et al., 2019). It has also been reported that BiP binds important functional partners such as co‐chaperones (HSP40 and HSP90) and UPR receptors (IRE1 and PERK) via its NBD (Kopp et al., 2019; Liu et al., 2020; Wang et al., 2022).

**FIGURE 1:**
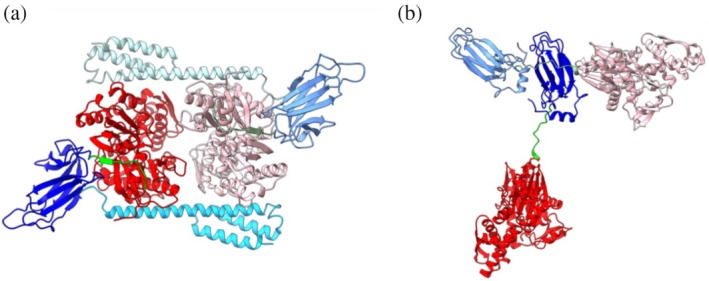
Two distinct immunoglobulin binding protein (BiP) dimer structures. (a) Crystal structure of human BiP dimer in ATP‐bound conformation (PDB 5E84) with nucleotide binding domain (NBD) in red, substrate binding domain (SBD) alpha (lid) in light blue, SBD beta in dark blue and the linker in green. Here the dimer formation is through interaction between the NBD of two BiP monomers. One monomer is colored lighter for more clarity. (b) Crystal structure of lid‐truncated hamster BiP dimer in apo conformation (PDB 7A4U) with the same color map as (a). Here the dimer formation is via the SBD of one BiP protomer and the linker of the other. Here the dimer formation is via the SBD of one BiP monomer and the linker of the other.

BiP is considered to function as a monomer, but has been shown to self‐associate into multiple oligomeric species that can affect its activity (Blond‐Elguindi et al., 1993; Carlino et al., 1992; Perera et al., 2019; Rivera, Burgos‐Bravo, et al., 2023; Tokunaga et al., 1992; Trcka et al., 2019; Yang et al., 2015). One proposed oligomer assembly is with an interface between the NBD of two BiP monomers (Figure 1a), from crystal structures of BiP with adenosine triphosphate (ATP) and adenosine monophosphate (AMP) (PDB: 5E84, 6ASY, 7N1R; Yang et al., 2015, 2017; Li et al., 2022). Another resolved structure involves binding between the SBD of one BiP protomeric unit and the linker of the other when BiP is in the apo or ADP‐bound states (Figure 1b, PDB: 7A4V, 7A4U—Preissler et al., 2020).

BiP has been shown to readily oligomerize in the absence of nucleotides or in the presence of ADP, but this behavior is reduced in the presence of ATP (Preissler et al., 2015; Rivera, Burgos‐Bravo, et al., 2023; Trcka et al., 2019). This can be explained, in part, by the fact that BiP NBD binding ATP provokes negative allosterism, diminishing the binding of polypeptide in the SBD (Huang et al., 2022). Interestingly, studies using non‐hydrolyzable ATPs such as ATPγS and adenylyl‐imidodiphosphate do not show clear patterns of how these ligands modulate BiP monomers binding to one another (Flynn et al., 1989; Liberek et al., 1991; Rivera, Burgos‐Bravo, et al., 2023). Together, all this information raises the question of whether BiP could have two binding sites for polypeptides, one in the SBD and another in the NBD.

Using the well‐characterized yeast BiP, this work focuses on determining the characteristics of BiP oligomerization in the presence or absence of nucleotides and peptides, using mass photometry (MP), an emerging single‐molecule method for quantifying protein monomer–dimer equilibria (Liebthal et al., 2021; Paul et al., 2022; Soltermann et al., 2020; Young et al., 2018). This includes an approach proposed here that allows real‐time measurement of protein‐binding kinetics. We also scrutinize the mechanical aspect of this reaction using nano‐rheology (NR) and enzyme activity assays. We show that dimer formation of BiP has two different affinities with orders of magnitude difference in the dissociation constant values and that ligands have different effects in the two affinity regimes.

## 2 MATERIALS AND METHODS

### 2.1 BiP expression and purification

Recombinant N‐terminal His‐tagged BiP from *Saccharomyces cerevisiae* was expressed in the RR1 *Escherichia coli* strain and purified by nickel affinity (General Electric, MA, USA) chromatography as previously described (Ramírez et al., 2017; Rivera, Mjaavatten, et al., 2023). Successful purification of concentrated BiP protein was confirmed by Coomassie blue‐stained 4%–20% lithium dodecyl sulfate‐polyacrylamide gel electrophoresis (LDS–PAGE) (Figure S1). In addition to the expected band for BiP at 72 kDa, a second band was visualized at around 60 kDa. This results from degradation of BiP during the purification process and constitutes less than 10% of the total protein content. The content of both bands was confirmed by mass spectrometry using trypsin digestion (Taplin Biological Mass Spectrometry Facility, Harvard Medical School, MA, USA). Protein concentration was determined using the Bradford assay with bovine serum albumin as a standard (Bradford, 1976), and percentages of purity were quantified using ImageJ software (Schneider et al., 2012). For BiP cysteine mutants for adhesion of protein to coverslips in NR studies, the work of Marcinowski et al. (2011) was considered, in which murine BiP V166C, G518C was used. Here, a corresponding *S. cerevisiae* BiP V185C, G537C BiP plasmid was engineered, and successful mutagenesis was verified by Sanger DNA sequencing (Sequetech, CA, USA). This substitution and subsequent protein purification and concentration were performed as outlined in Casanova‐Morales et al. (2018).

### 2.2 Peptide selection and synthesis

For the BiP SBD client, an eight‐amino‐acid‐long peptide substrate PP28 previously shown to bind to BiP (Blond‐Elguindi, 1993; Pobre et al., 2019) was selected. PP28 weighs 1144 Da and has the amino acid sequence HWDFAWPW. This complies with the BiP binding sequence Hy‐W‐X‐Hy‐X‐Hy‐X, whereby Hy is any hydrophobic amino acid, W is tryptophan, and X is any amino acid (Knarr et al., 1995). The small size of this molecule ensures that it remains unfolded in solution to interact with BiP and does not contribute any mass value to interfere with MP measurements of BiP assemblies. The peptide NP53 (sequence AGEYYAAL) weighing 857 Da, previously shown to have no binding activity to BiP (Blond‐Elguindi et al., 1993), was employed as a negative control. Both peptides were manufactured to >95% purity by BioServUK, Rotheram, UK.

### 2.3 Mass photometry studies

Prior to MP measurement, all reactions were equilibrated at room temperature in a buffer solution composed of 20 mM HEPES pH 7.4 (H3375, Merck Life Science UK Limited, Dorset, UK) 200 mM KCl (0395, Amresco, PA, USA), 0.1 mM ethylenedinitrilotetraacetic acid (E5134, Merck Life Science UK Limited. Dorset, UK), and 2 mM MgCl_2_ (M2670, Merck Life Science UK Limited, Dorset, UK).

MP measurements were all conducted using a commercially available mass photometer (TwoMP, Refeyn Ltd., Oxford, UK). Microscope glass coverslips (24 × 50 mm, Menzel Gläser 630‐2603, VWR, Leicestershire, UK) were thoroughly cleaned via three sequential cycles of 5‐min bath sonication, first with Milli‐Q® water (18.2 MΩ cm) (Milli‐Q), followed by 50% isopropanol in Milli‐Q, and a final rinse in Milli‐Q. Coverslips were then dried using nitrogen flow. Three millimeter silicone gaskets (GBL103250, Grace Bio‐Labs, OR, USA) were affixed to the coverslip surface. Around 30 μL Immersion oil is then dropped onto mass photometer objective and the coverslip placed on the mass photometer stage. The gasket was then prefilled with the equilibration buffer or PBS (MBD0058, Sigma Aldrich, Dorset, UK), and the focus was adjusted accordingly before calibrant or sample measurement. Calibration of the contrast to mass followed the methodology outlined in Foley et al. (2021).

Immediately before each measurement, the equilibrated reaction mixture was either rapidly diluted prior to loading onto the gasket or loaded directly on top of the preloaded buffer by gentle pipette mixing to achieve a final measurement concentration of 10 nM BiP in 20 μL. For reactions containing 10 nM or less of BiP, preloaded buffer in the gasket was completely removed and replaced with 20 μL of equilibrated reaction mixture.

The recording of a 1‐min video commenced promptly after the addition of the protein sample, capturing the landing events of individual proteins on the coverslip surface. This protocol was repeated a minimum of three times for each solution condition. All recordings were captured utilizing a field of view measuring 10.9 by 4.3 μm, operating at a frame rate of 500 Hz, which was subsequently subjected to frame binning at a factor of 2, resulting in an effective frame rate of 250 Hz. Analysis of the recorded videos was carried out using DiscoverMP software (Refeyn Ltd., Oxford, UK), producing continuous ratiometric sequences with a rolling average window set at 12 frames (equivalent to 48 ms). Default threshold parameters for particle identification were employed, specifically 1.5 (threshold 1) and 0.25 (threshold 2).

Mass histograms were individually inspected and analyzed using Refeyn Discover MP software v2023 R1.2. We ensured negligible unbinding of BiP from glass surface in MP was observed in our experimental conditions (Figure S5). Gaussian curve fittings were manually examined to avoid the inclusion of low‐mass noise contribution to the peaks. Monomer, dimer, trimer, and tetramer peaks were considered those around 72, 144, 216, and 288 kDa, respectively, ±5% as per instrument specifications. To verify the ability of BiP to adhere to the glass surface and to ensure accurate counting of protein molecules, we observed the unbinding events across BiP concentrations (Figure S2). To check for species detection bias or disassociation effects during measurement we examined the variation in the oligomeric state of BiP over the course of the 1 min data acquisition (see Figure S3). Results outlined here focused on dimer fraction counts, normalized by expressing the dimer‐to‐monomer ratio (D:M). Data fitting and statistical analysis were performed using GraphPad version 8.0.2.

#### 2.3.1 BiP dimerization at different concentrations

In the case of studies into concentration dependency of BiP dimerization, purified BiP in solution (buffer described above) at given concentrations was incubated at room temperature for a minimum of 15 min. MP measurements were then taken and acquired D:M values plotted against the untransformed or log_10_ concentration values of BiP incubation. To obtain *K*_D_ values, curves were fitted to data using either a monophasic hyperbole equation:

$$y=B_{\max}*\frac{x}{\;K_{D}+x}.$$

Or a biphasic hyperbole equation:

$$y=B_{1,\max}*\frac{\;x}{K_{D,1}+x}+B_{2,\max}*\frac{\;x}{K_{D,2}+x}.$$

Akaike information criteria were then used to choose the best of the two models (Akaike, 1998), see Supporting Information S1 for more details. Impact of higher‐order oligomer formation was also scrutinized (see Figures S4 and S5).

#### 2.3.2 BiP dimerization kinetics

For BiP dimer dissociation rates (*k*_off_ values), 120 μM of BiP in solution was diluted to 100 nM, and MP measurement was performed immediately to establish a time (*T*) = 0 point. This reaction was then left to incubate at room temperature and MP measurements taken at time intervals until the D:M ratio plateau was reached. The dimer prevalence was then plotted as a function of time, and the *k*_off_ determined via fitting a one‐phase exponential decay to the data with the equation:

$$y=\left(\;y0-\text{Plateau}\right)*\exp\left(-K*x\right)+\text{Plateau}.$$

In association kinetic studies, 2 mM ATP (A26209, Merck Life Science UK Limited) was added to 120 μM BiP and incubated at room temperature, and MP measurements taken immediately and at time intervals until the plateau of D:M value reached. Data were then treated to consider the true time = 0 point as when all ATP was hydrolyzed to ADP by BiP (as confirmed by ATPase activity studies, detailed below). The dimer prevalence was plotted as a function of time and *k*_on_ values extrapolated by the fitting of a one phase association to the data using the equation:

$$y=y0+\left(\text{Plateau}-y0\right)*\left(1-\exp\left(-K*x\right)\right).$$

#### 2.3.3 BiP dimerization in the presence of ligands

To investigate the impact of different concentrations of ligand (peptides or nucleotides) on BiP dimerization, 100 nM of BiP was incubated at room temperature with ligand at given concentration for a minimum of 15 min before MP measurements were taken. D:M values were then plotted against ligand concentration and fitted to the one phase dissociation curve (above).

For studies into the effect of ligands upon different concentrations of BiP, peptides (PP28 and NP53) at 5 μM or nucleotides (ATP, ADP, or adenosine 5′‐O‐(3‐thio)triphosphate (ATPγS) [Thermo Scientific, MA, USA]) at 2 mM were incubated with 100 nM (constituting a low concentration) or 120 μM (a high concentration) of BiP for a minimum of 45 min. A minimum of three MP measurements were then taken for each condition, D:M ratios calculated, and mean and standard error values reported.

### 2.4 Nano‐rheology studies

NR is a novel ensemble technique that allows direct measurement of mechanical properties of enzymes in their folded states (Alavi et al., 2015; Casanova‐Morales et al., 2018). In this technique, the enzyme under study tethers gold nanoparticles to a gold surface. An oscillatory voltage then drives the negatively charged gold nanoparticle, hence applying a mechanical force on the enzyme (see Supporting Information S1 for detailed explanation of technique and Figure S7 for instrument set‐up). The frequency response of the enzyme, which is the amplitude of its deformation under the force, is then measured using evanescent wave microscopy. By measuring the frequency response of an enzyme at different concentrations of a ligand, one can obtain binding curves and deduce the dissociation constant (*K*_D_). In this study, we employed NR to investigate the effect of peptide (PP28) binding on BiP's mechanical properties in two conditions: when ATP is present and when it is not. The amplitude of deformation of BiP was measured at different concentrations of PP28, with or without 2 mM of ATP. Amplitude values were then plotted as a function of PP28 concentration in GraphPad 8.0.2 and fit to either one‐phase exponential decay or hyperbolic equations (above). *K*_D_ values were taken from these fittings and a one tailed *t*‐test with Welch's correction (Welch, 1947) was performed to compare the two conditions.

### 2.5 Activity assays

The enzymatic functionality of BiP protein, specifically its ATPase activity leading to ADP production, was evaluated by monitoring changes in absorbance at 340 nm, indicative of nicotinamide adenine dinucleotide (NADH) oxidation to NAD^+^ (McFarlane & Murray, 2020). This assay comprises three consecutive reactions driven by BiP's catalytic function, modulated by K^+^ and Mg^2+^ (McFarlane & Murray, 2020). Initially, ATP hydrolysis occurs, yielding ADP and Pi. This is coupled to pyruvate kinase (PK) and lactate dehydrogenase (LDH), present at non‐saturating concentrations. PK utilizes ADP and phosphoenolpyruvate (PeP) to replenish ATP, ensuring ATP availability isn't a limiting factor in the coupled reaction. LDH facilitates the conversion of pyruvate to lactate, concurrently producing NAD^+^ via oxidation of oxidizing NADH. Reaction set‐up was: 120 μM BiP, 2 mM ATP, 50 mM HEPES at pH 7.4, 125 mM KCl (Merck), 2 mM MgCl2 (Merck), 400 μM PeP (Sigma), 300 μM NADH (Sigma), along with 2 Units of PK (Roche, IN, USA) and LDH (Sigma) auxiliary enzymes, and peptide PP28 at varying concentrations, in a final volume of 200 μL. The absorbance measurements were performed at 20 and 30°C on a Nanoquant Infinite M200PRO spectrophotometer (Tecan, Männedorf, Switzerland) taken at 60 s intervals for 50 min. Given the 1:1 ratio of NADH to ATP, the extrapolated data were employed to derive ATP concentration values over time. ATP concentration data were then plotted against respective time in seconds in GraphPad Prism 8.0.2 and linear regression analysis performed to observe time point of ATP depletion.

### 2.6 BiP cross‐linking

First, 45 μM of BiP sample underwent buffer exchange to phosphate buffered saline (PBS) and incubated at room temperature with or without 2 mM ATP and 2 mM M MgCl_2_ for 10 min. Cross‐linking of these samples was then performed by incubation of each with 1 mM disuccinimidyl sulfoxide (Pierce, TX, USA) for 30 min to allow cross‐linking of primary amine groups (as per Kao et al., 2011). The size of constructs within these samples were then interrogated by MP and gel electrophoresis. Electrophoresis results were then quantified by measuring intensity density values using Fiji image analysis software (Schindelin et al., 2009). These data were then normalized to a background measurement and dimer, trimer, and tetramer values calculated as a proportion of the monomer band.

## 3 RESULTS

### 3.1 BiP oligomerization is concentration‐dependent and biphasic

**FIGURE 2:**
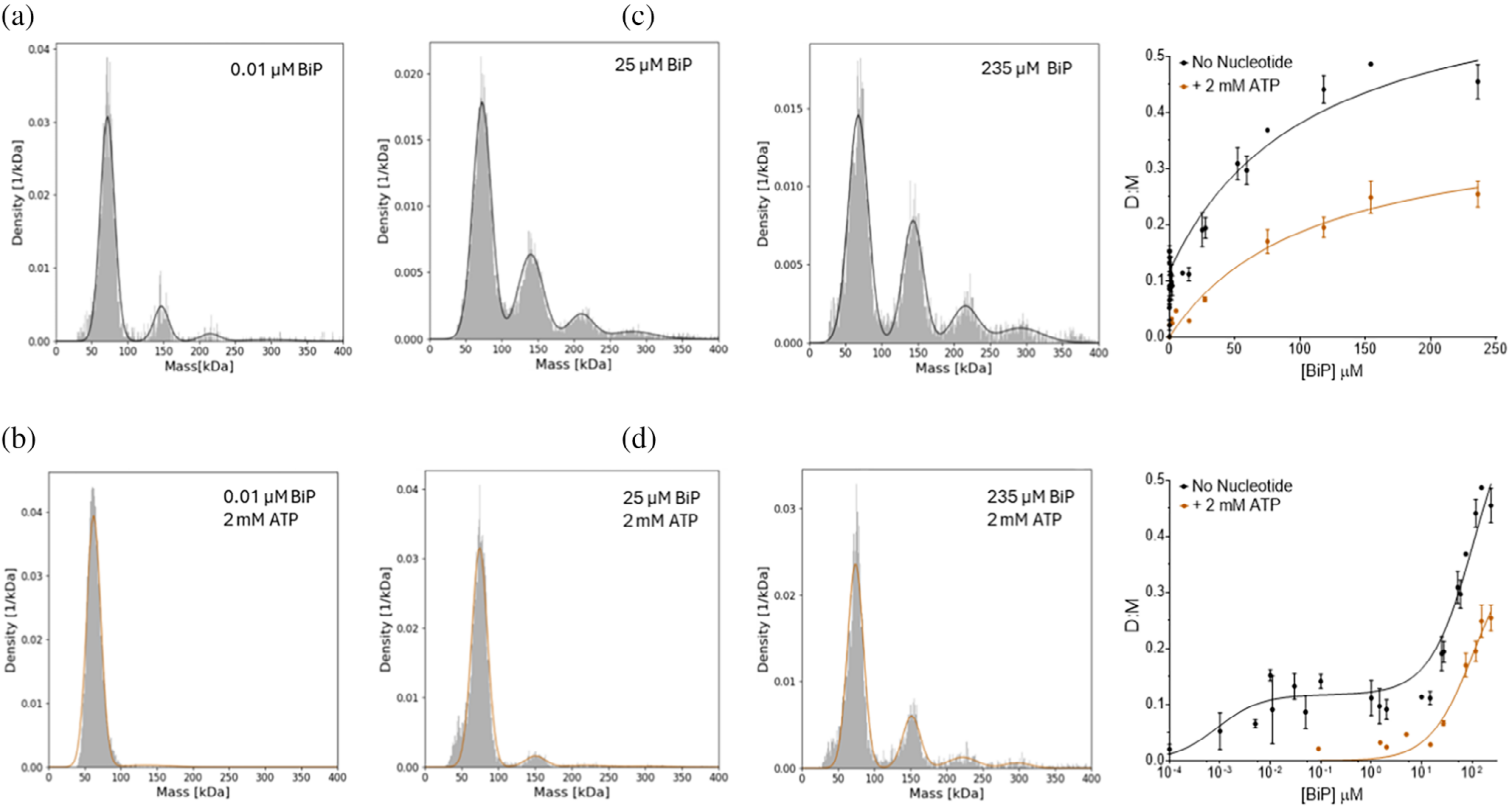
Immunoglobulin binding protein (BiP) oligomerizes in a concentration and ATP‐dependent manner. (a, b) Representative mass photometry histograms and Gaussian fittings of BiP at 0.01, 25, and 235 μM in the absence (a) and presence (b) of 2 mM ATP. (c) BiP dimer to monomer ratio (D:M) as a function of total BiP concentration (on a linear scale) in the absence (black data, biphasic hyperbole line fit) and presence (orange data, monophasic hyperbole line fit) of 2 mM ATP. Symbols and error bars represent the mean values and standard errors of at least three technical repeats. (d) The same data sets and fitting as (c) with total BiP concentration on the log10 scale, emphasizing the biphasic relationship.

To understand the existence of BiP oligomer states, we used MP to quantify the relative abundance of monomers and dimers across a range of protein concentrations (Figure 2b). In agreement with our previous work (Rivera et al., 2023), the monomer (measuring 72 ± 4 kDa) was the dominant species across all concentrations with dimers (144 ± 5 kDa) as the second most abundant species. At concentrations of 10 nM and above, trimers, tetramers, and higher‐order species were also detected (Figures 2b, S4B, and S5). To derive a *K*_D_ value for the BiP monomer/dimer equilibrium, the dimer‐to‐monomer (D:M) ratios were plotted against total BiP concentration. Here, we observed two plateaus in the D:M ratio: the first at low BiP concentrations (around 0.1 nM to 5 μM), and a second at high BiP concentrations (around 100–230 μM). Thus, we fitted the data with a biphasic hyperbole curve to determine the dissociation constants for the two binding events. The biphasic curve fit the data better than monophasic fitting (verified by Akaike information criteria, see Supporting Information S1) with two statistical plateaus at D:M values of 0.12 ± 0.01 and 0.55 ± 0.08, and *K*_D1_ and *K*_D2_ of 0.009 ± 0.001 and 108.3 ± 34.2 μM, respectively (Figure 2a,b and Table 1).

**TABLE 1:** Immunoglobulin binding protein (BiP) dimer dissociation constants and plateau values.

BiP dimer	ATP	*K*_D_ (μM)	Plateau
High affinity	No	0.009 ± 0.001	0.12 ± 0.01
Low affinity	No	108.3 ± 34.2	0.55 ± 0.08
Low affinity	2 mM	108.7 ± 42	0.39 ± 0.01

*K*_D_ and *B*_max_ values as fitted to mass photometry results. Reported values are statistical means ± standard error of at least three technical repeats.

### 3.2 ATP reduces dimer formation—particularly at lower concentrations

Considering previous reports of ATP reducing BiP oligomer formation (Carlino et al., 1992; Trcka et al., 2019), we repeated the experiment above in the presence of 2 mM ATP across all BiP concentrations (Figure 2b). We detect a clear reduction in the abundance of dimer and higher‐order species, particularly pronounced at low (nM to low μM) BiP concentrations. In contrast to the above, variation in the D:M ratio with BiP concentration is best fit with a monophasic hyperbole with a *K*_D_ value of 108.7 ± 42 μM. This also demonstrates that the high‐affinity (low *K*_D_) dimer is the form most affected by ATP, reflected in the similarity of *K*_D_ values (Figure 2a,b and Table 1).

### 3.3 BiP dimer dissociation is faster in the presence of ATP

**FIGURE 3:**
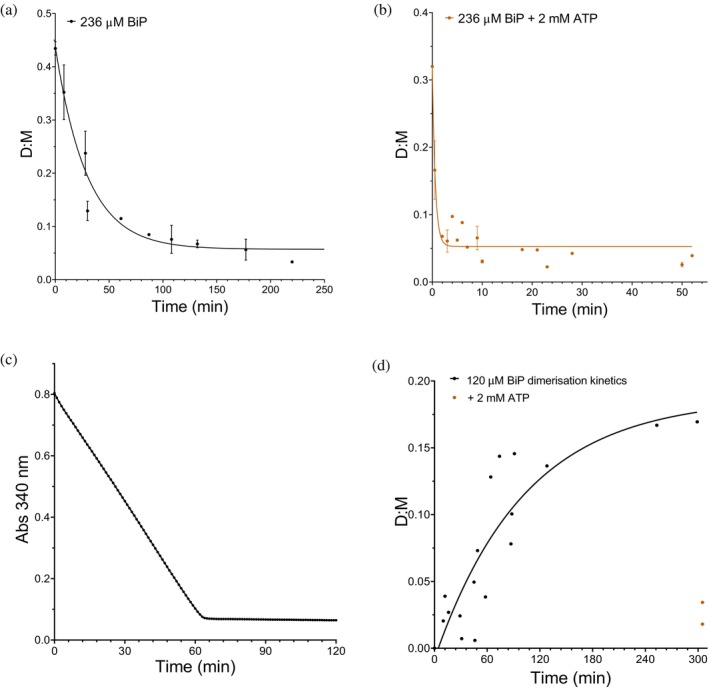
Immunoglobulin binding protein (BiP) dimerization kinetics. (a) BiP D:M of 235 μM BiP diluted to 100 nM (mean points with standard error of mean from at least three technical repeats) plotted against time and the one phasic decay equation line fit to data shows BiP dimer dissociation relatively slow. (b) This same BiP dimer dissociation in the presence of 2 mM ATP is a much faster reaction as ATP catalyzes BiP monomerization. (c) ATPase activity assays reveal that 235 μM BiP fully converts 2 mM ATP to ADP after 60 min. (d) The increase of dimer formation following the conversion of ATP to ADP plotted as a function of time allows the one phase association curve to be fit to the data and the kinetics derived. At point of plateau, 2 mM ATP added to reaction as means of control to demonstrate recovery of BiP monomers (orange points).

To further characterize BiP dimerization, we assessed the kinetics of this reaction via MP approaches. When D:M values of BiP alone are plotted as a function of incubation time (0–260 min), the *k*_off_ value of BiP dimers at 0.1 μM is determined as 0.03 min^−1^ (Figure 3a). When this protocol is repeated in the presence of 2 mM ATP, the BiP monomerization reaction is catalyzed, increasing the *k*_off_ value to 3.8 min^−1^ (Figure 3b).

### 3.4 MP measurement of BiP dimer association kinetics via novel protocol

As a method to explore rates of dimer association, we leveraged the ATPase activity of BiP and the fact that ATP favors BiP monomerization, whereas the presence of ADP promotes dimer formation (Preissler et al., 2015; Rivera, Burgos‐Bravo, et al., 2023; Trcka et al., 2019). Initially, 2 mM ATP was added to 120 μM BiP to favor the monomeric state. The sample was then incubated at room temperature, during which BiP gradually hydrolyzed ATP to ADP. As ADP accumulates, dimer formation becomes increasingly favored. MP measurements were taken at defined time intervals (from 0 to 290 min) to monitor changes in the D:M values as the nucleotide composition of the reaction shifted over time.

To confirm the timescale of ATP conversion to ADP by BiP at room temperature, ATPase activity assays were performed. From these, the lifetime of 2 mM ATP in solution with 120 μM BiP was calculated to be approximately 60 min (Figure 3c). After this time, ADP will be the dominant nucleotide species in the reaction and BiP dimer formation favored. Thus, MP data were corrected so the time point at 60 min and corresponding D:M value (0.28) became the new time point at 0 min and D:M = 0 by subtracting all time and D:M values by 60 and 0.28, respectively (plot of non‐treated data shown in Figure S6). These new origin values represent the point at which ATP is fully hydrolyzed and ADP begins to promote dimer formation. From this, the apparent association rate constant (*k*_on_) for BiP dimer formation was determined to be 0.01 μM^−1^ min^−1^ (Figure 3d).

To validate this ATPase‐based system as a method to study dimerization kinetics, 2 mM ATP was added after dimer formation had reached a plateau. This led to a reversal of dimerization and restoration of the D:M to pre‐ADP levels (Figure 3d). This result supports the interpretation that the observed increase in dimerization is driven by the decrease of ATP over time. This constitutes a novel method for probing association kinetics using MP, and this framework could be adapted to study *k*_on_ values in other protein–protein interactions.

### 3.5 MP measurement of small ligand binding via monitoring dimerization

**FIGURE 4:**
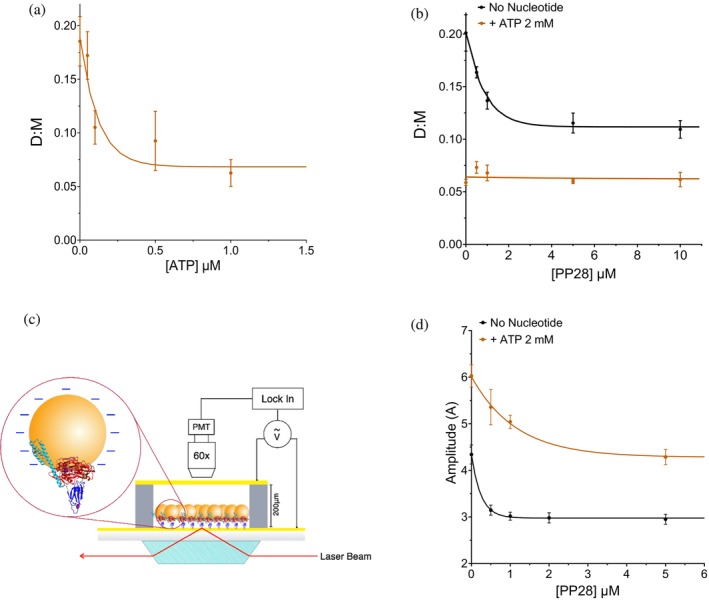
Monitoring dimer formation gives insight to protein‐ligand interactions. (a) Dimer formation of 0.1 μM immunoglobulin binding protein (BiP) observed as a function of ATP concentration via mass photometry reveals a *K*_D_ value of 0.8 μM. (b) Dimer formation of 0.11 μM BiP observed as a function of peptide substrate concentration via mass photometry reveals a *K*_D_ value of 0.6 μM. When this is performed in the presence of 2 mM ATP, no equilibrium parameters can be derived as it appears that the dimer presence is reduced to maximal levels at 0 μM peptide. (c) Nano‐rheology experimental set‐up demonstrating the positions of cystine mutations in BiP to tether protein to the gold surfaces. BiP structure was obtained from Alphafold using yeast BiP sequence. Cysteine mutations are shown in purple, nucleotide binding domain (NBD) in red, substrate binding domain (SBD) alpha (lid) in light blue, SBD beta in dark blue and the linker in green. (d) Nano‐rheology studies performed as a function of peptide in the presence and absence of 2 mM ATP reveal *K*_D_ values of 0.6 and 1.2 μM, respectively. All points plotted represent mean values with standard error of mean from at least three technical repeats, and all line fittings are from the one phase decay equation.

Following the above results, we wanted to further probe the observed BiP monomerization by ATP using MP. To achieve this, dimerization of 100 nM of BiP was studied as a function of ATP concentration. The rise in relative monomer prevalence with increased ATP concentration confirms that this effect is indeed nucleotide‐dependent (Figure 4a). Here we see the statistically derived plateau of dimer formation as D:M = 0.05 *±* 0.005, reached by ~1 μM of ATP (Figure 4a). From the fitting of this data to exponential decay curves, the *K*_D_ of ATP and BiP was derived as 0.3 ± 0.1 μM. *K*_D_ values eluded here are in the same order of magnitude as those previously reported from a range of techniques (Table S3).

To examine the effect of SBD occupancy on BiP oligomerization, PP28, a hydrophobic 8‐amino‐acid peptide reflecting the BiP SBD binding motif and previously demonstrated to bind to BiP, was employed (Blond‐Elguindi, 1993; Knarr et al., 1995).

The effect of PP28 on BiP D:M was investigated in the presence and absence of 2 mM ATP. In the case of PP28 alone, a concentration‐dependent decrease in BiP dimerization is seen before a D:M plateau of 0.10 ± 0.01 is obtained by 5 μM of PP28 (Figure 4b). This result demonstrates that SBD occupancy by peptide inhibits BiP–BiP binding, but not to the same degree as ATP. In the presence of ATP, PP28 concentration studies into BiP dimer formation show a reduction of D:M values to 0.05 ± 0.003, as in the case of ATP alone and no further reduction is seen with increasing concentration of peptide (Figure 4b).

We also derived the *K*_D_ of PP28 with BiP as 1.0 ± 0.04 μM. To corroborate this result from novel MP studies, we performed NR studies of BiP in the presence of a range of concentrations of PP28 (Figure 4d), in which a *K*_D_ value of PP28 with BiP was calculated as 1.54 ± 0.75 μM, similar to those seen in MP. From the NR studies, we were also able to derive the *K*_D_ value of PP28 in the presence of ATP as 0.78 ± 0.55 μM, exhibiting the expected reduction of BiP SBD affinity for peptide in the ATP‐bound state. NR studies also allowed insight into the mechanical properties of BiP in the presence of these ligands. Here we observe that BiP is stiffer with PP28 alone in comparison to ATP and PP28, reflecting previously reported NR findings that ATP binding makes BiP softer in terms of viscoelasticity (Casanova‐Morales et al., 2018).

These results also verify a novel approach to assessing small ligand interactions with proteins via MP, in which binding parameters can be eluded via monitoring of oligomerization or changes in molecular weight.

### 3.6 BiP concentration dictates the effect of ligands on dimerization

To characterize how BiP oligomerization is impacted by the library of ligands in this system, dimerization measurements were taken following incubation of 100 nM or 120 μM of BiP with either 2 mM of ATP, ADP, or ATPγS, or 5 μM of PP28, or a control “scrambled” peptide NP53.

**FIGURE 5:**
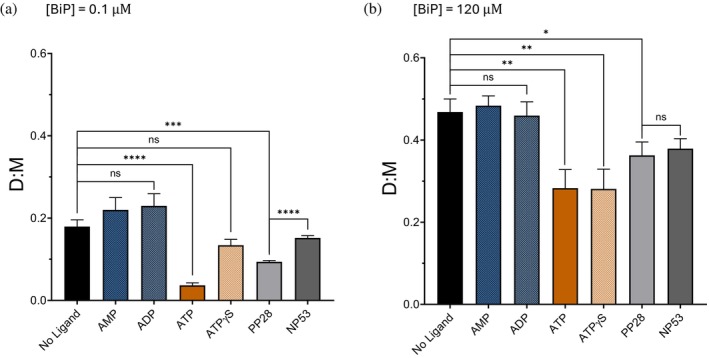
Immunoglobulin binding protein (BiP) dimer formation exhibits concentration‐dependent responses to ligands. (a) In nanomolar range concentrations ATP and peptide substrates reduce dimer assembly with ATP having the greatest effect. Other ligands (including scrambled control peptide) do not significantly alter BiP dimer formation at this concentration. (b) At micromolar concentrations of BiP, peptide does not impact dimer prevalence but ATPγS reduces the dimerization to the same degree as ATP. ATP and ATPγS are the only ligands that significantly impact BiP dimer formation at this concentration. Also, ATP perturbation of BiP dimer at this concentration is much less pronounced than at nanomolar concentrations. Mean D:M values and standard errors from at least three technical repeats are reported. ns = *p* > 0.05, *p ≤ 0.05, **p ≤ 0.01, ***p ≤ 0.001 ****p ≤ 0.0001.

MP reveals that in 100 nM conditions BiP D:M value in the absence of nucleotides (0.18 ± 0.06) is comparable to those in the presence of ADP or ATPγS (0.23 ± 0.06 and 0.13 ± 0.03, respectively). However, with ATP, D:M falls significantly (0.03 ± 0.02). This dimerization was also reduced by PP28 presence but only to the value of 0.1 ± 0.005, and importantly, no significant change was observed in the case of the NP53 control peptide (Figure 5a).

This profile changes when a high concentration of 120 μM BiP is used. In this case, the only two ligands that alter BiP dimerization are ATP and ATPγS, both reducing D:M by 40% from 0.46 ± 0.06 to 0.28 ± 0.05 (Figure 5b).

At low concentrations of BiP, ATP decreases dimerization but ADP or ATPγS do not. ADP and ATPγS have either an absent or modified gamma phosphate group. This chemical group is essential for interactions with lysine 70 of the BiP NBD to initiate negative allosteric communication to protein substrate binding in the SBD via proline 147 (Kityk et al., 2012; Qi et al., 2013; Vogel et al., 2006). This suggests that a dimer that forms at low BiP concentrations is inhibited by ATP via this communication to the SBD. At high BiP concentrations, ATPγS exhibits the same effects as ATP to indicate the presence of a dimer species that is influenced by the presence of nucleotide backbone occupation of the NBD but not the negative allosteric effect it has on the SBD.

### 3.7 BiP cross‐linking stabilizes dimers and higher‐order species

**FIGURE 6:**
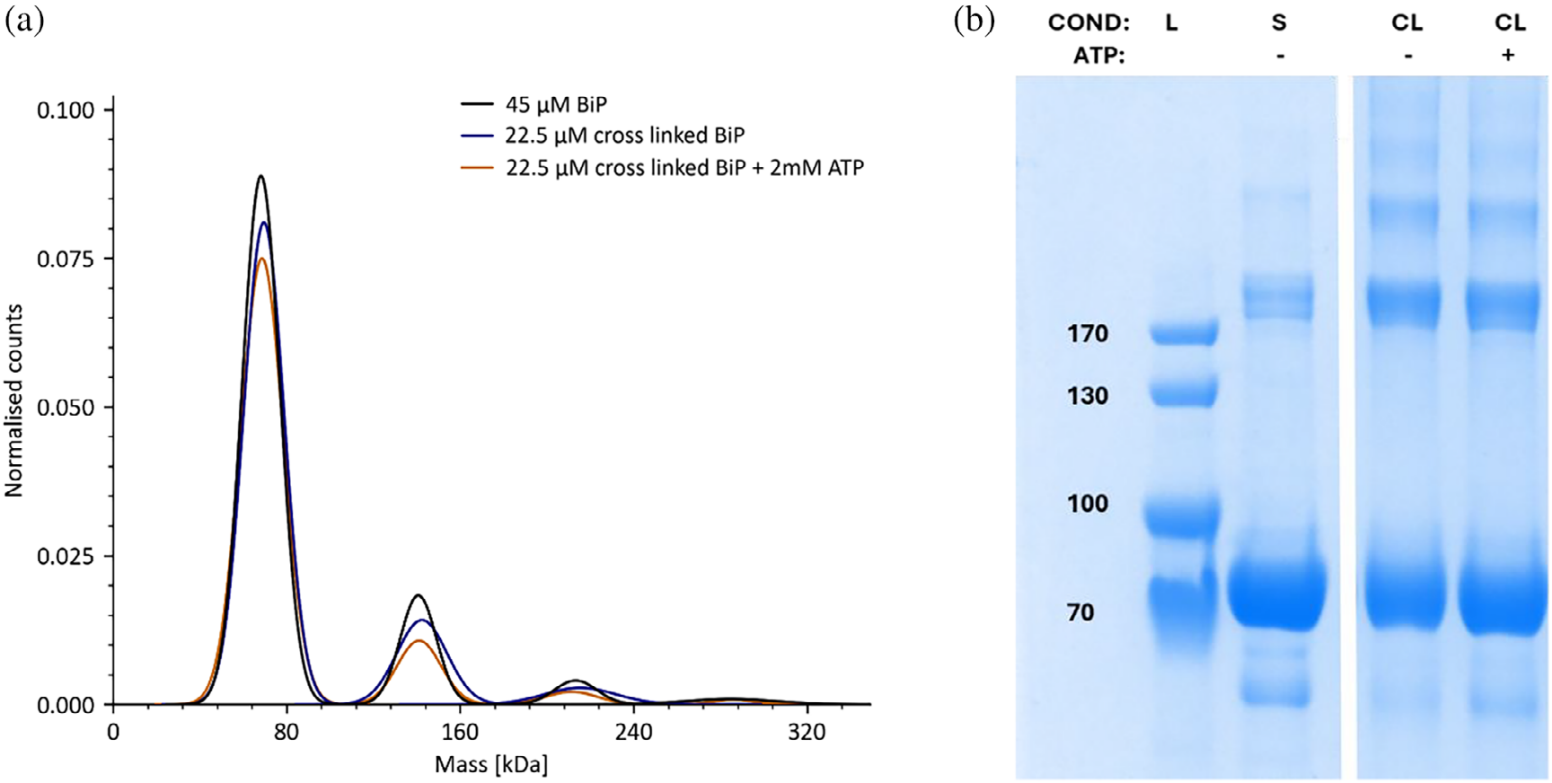
Cross‐linking studies of immunoglobulin binding protein (BiP). (a) Mass photometry Gaussian fitting of normalized counts of BiP species in solution with 45 μM non‐cross‐linked or 22.5 μM cross‐linked sample in the presence or absence of 2 mM ATP. We see that these conditions produce similar results to suggest fortified dimerization following cross‐linking and the resistance of this to monomerization effects of ATP. (b) LDS–PAGE gel of BiP in solution, and BiP cross‐linked in the presence and absence of ATP stained with Coomassie blue solution. Conditions: L = ladder (Invitrogen 26630), S = BiP in solution, CL = cross‐linked BiP. This shows cross‐linking leads to a reduced monomer band and increased intensity of bands at higher molecular weights. ATP addition to cross‐linking reactions does not seem to perturb oligomers.

Cross‐linked BiP was measured via MP and compared to non‐treated BiP in solution. Here, the D:M values of 22.5 μM cross‐linked BiP, half the concentration of the 45 μM non‐cross‐linked sample, were both 0.22 (Figure 5a). When ATP was included in the cross‐linking reaction, MP revealed a nominal decrease in dimer prevalence of just 0.07 (0.22–0.15) and a trimer reduction of 0.025 (0.06–0.035) (Figure 6a).

Visualization of both non‐cross‐linked and cross‐linked BiP samples via LDS–PAGE clearly revealed the presence of BiP monomers at the expected molecular weight of ~70 kDa. The next largest band size observed in all samples corresponds to 170 kDa of the protein standard. Considering the outcomes of MP studies on these samples, we infer that these bands are indeed the BiP dimer constructs that migrated through the gel at a slower rate than expected. This could be due to incomplete denaturation or dimerization via cysteines of the sample prior to loading, also explaining why dimers are seen in non‐cross‐linked BiP samples as some non‐covalent bonds between protomers remain (Hames, 1998). Interestingly, two bands are seen around the same size as the suspected dimer—which could also be explained by constructs of the same size but distinct conformations that had not undergone full denaturation.

Functionality of the cross‐linking reaction was confirmed as an increase in intensity of the dimer band, appearance of higher molecular weight bands, and the compensatory decrease in monomer band intensity. When the band intensities were quantified and non‐cross‐linked and cross‐linked BiP samples compared, dimer presence as a fraction of monomers was seen to increase by 38% (from 0.23 ± 0.02 to 0.61 ± 0.06), trimers by 23% (from 0.03 ± 0.009 to 0.26 ± 0.02), and tetramers by 14% (from 0 to 0.14 ± 0.03) (Figure 6b, Lane 4).

This oligomer presence did not differ greatly when the BiP cross‐linking reaction was performed in the presence of 2 mM ATP. Here, dimer, trimer, and tetramer presence relative to monomers were seen to increase by 27%, 17%, and 10% (to 0.5 ± 0.04, 0.2 ± 0.01, and 0.11 ± 0.03), respectively (Figure 6b, Lane 5). This represents just an 11%, 6%, and 4% decrease in BiP cross‐linked dimer, trimer, and tetramer formation when ATP was present.

## 4 DISCUSSION

### 4.1 BiP forms two dimers with distinct affinities

Previous studies have demonstrated that BiP dimerizes in a concentration‐dependent manner, with dimer formation increasing as concentration rises (Rivera, Burgos‐Bravo, et al., 2023; Sun et al., 2019). Using MP, we reveal that this relationship is biphasic, with two distinct *K*_D_ values spanning different orders of magnitude. Our data suggest the existence of two separate BiP dimeric forms. At nanomolar concentrations, a “high‐affinity” dimer predominates, while at micromolar concentrations, a “low‐affinity” dimer becomes more prevalent (Figure 2).

The high‐affinity, low‐*K*_D_ dimer is more sensitive to ATP‐mediated dissociation than the high‐*K*_D_ form. This is shown simply by ligand effects on BiP dimer prevalence at different BiP concentrations. At 100 nM, dimerization is significantly disrupted by ATP and (to a lesser extent) the peptide PP28 (Figure 4a), thus, one BiP protomer is binding to the other in a similar manner to unfolded protein client. This suggests that the low‐*K*_D_ dimer forms via interactions in the SBD, aligning with the oligomeric structure proposed by Preissler et al. (2020) (Figure 1b), in which one BiP subunit's linker binds into the SBD of another. In this high‐affinity (low *K*_D_) dimer ATP reduces dimerization allosterically, while PP28 likely blocks dimer formation through steric hindrance in the SBD.

The differential effect of ATP on BiP dimer forms is also shown by cross‐linking studies. Only a modest ATP‐mediated reduction in dimer levels at μM BiP concentrations is seen when cross‐linking was performed, that is, low‐affinity dimers were resistant to ATP‐mediated monomerization (Figure 5). The appearance of two bands for BiP dimers in non‐cross‐linked LDS gels also supports the presence of separate dimer types. Since electrophoresis does not involve dilution, these observations independently validify our MP results, which do include dilution steps.

At higher BiP concentrations (120 μM), PP28 causes only a minor, non‐significant decrease in dimerization. This suggests that while the high affinity (low *K*_D_), SBD‐linker dimer may be inhibited, the low‐affinity (high *K*_D_) dimer dominates at these concentrations and likely forms independently of the SBD. Moreover, ATP and ATPγS reduce low‐affinity (high *K*_D_) dimer levels equally, indicating that in this form the negative allosteric communication to the SBD is not central to its de‐stabilization. Instead, local influences of NBD occupancy may interrupt a dimerization site in this domain. These effects are not as pronounced as the allosteric effect and could represent an ATP‐bound dimer. Recent high‐resolution kinetic analysis of BiP's NBD (Mas & Hiller, 2025) revealed that nucleotide binding and hydrolysis induce defined conformational transitions within the NBD, with long‐lived ATP‐, ADP·Pi‐, and ADP‐bound states. These transitions occur on the timescale of tens of seconds, suggesting that nucleotide occupancy alone can transiently reorganize the NBD surface independently of SBD coupling. These slow, nucleotide‐dependent transitions could underlie the subtle, local effects of ATP binding we observe on high‐*K*_D_ dimerization, suggesting that this dimer form may reflect an ATP‐occupied or hydrolysis‐competent state rather than one governed by SBD allostery.

Crystal structures of ATP‐bound BiP dimers reveal direct NBD–NBD interactions (Li et al., 2022; Yang et al., 2015, 2017). These ATP‐bound BiP dimers do not bind substrate peptides (Yang et al., 2017), supporting our observation that peptides do not affect low‐affinity dimerization (Figure 5). Li et al. (2022) further showed that this dimer can hydrolyze ATP directly to AMP. While our current data show no significant effects of AMP or ADP on dimer levels, further MP studies could explore this.

Though low‐affinity dimer formation may involve ATP‐induced lid opening in the SBD, our findings do not suggest that ATP binding is required. Molecular dynamics studies have shown lid opening in the apo state (Mahto et al., 2024), implying that this conformation—and thus dimerization—may occur in the absence of nucleotide. This supports the idea that BiP oligomerization is an inherent feature of its regulatory mechanism.

**FIGURE 7:**
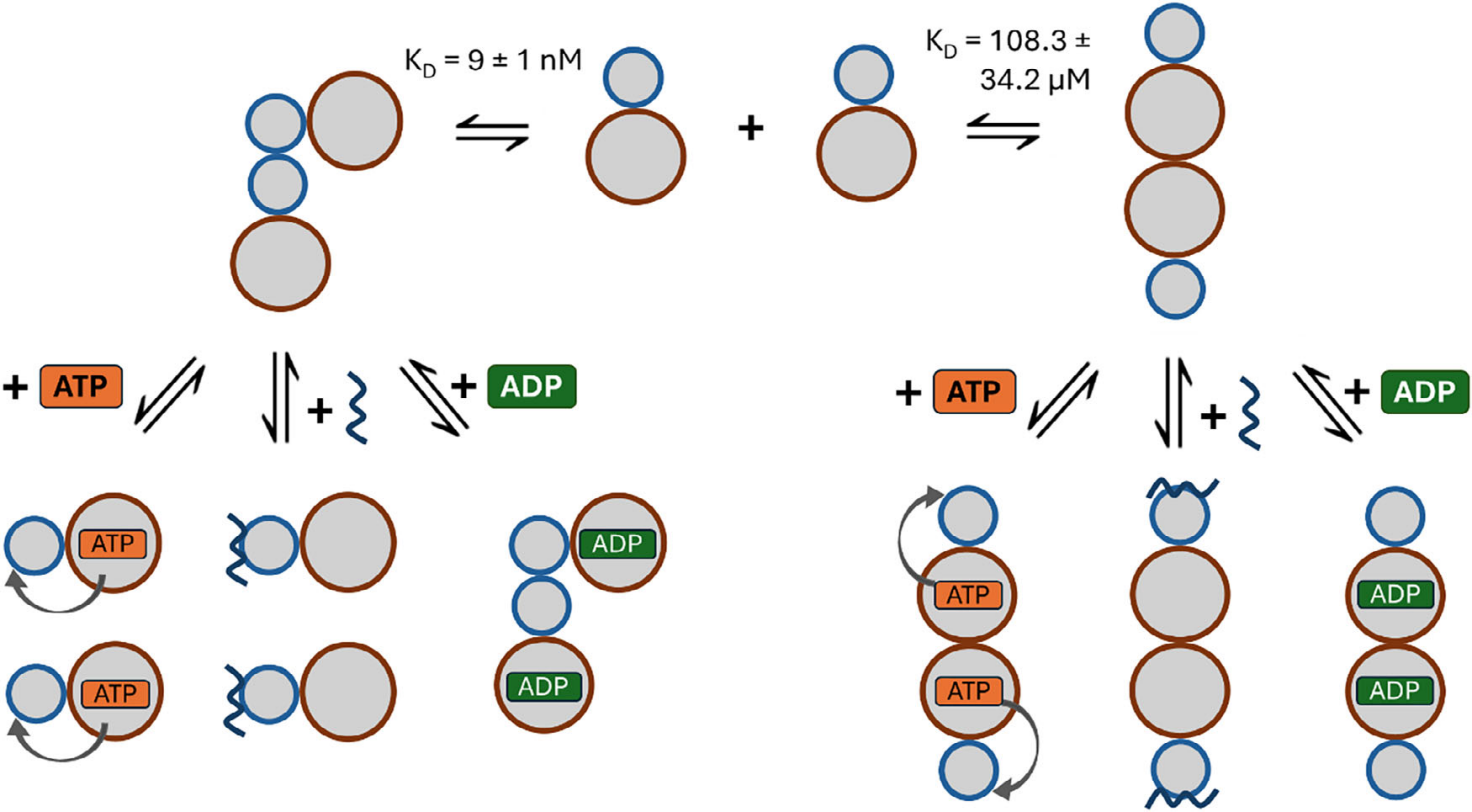
Schematic of the proposed model of immunoglobulin binding protein (BiP) dimerization. BiP monomers are shown as light or dark gray circles with the larger (orange outline) nucleotide binding domain (NBD) and smaller (blue outline) substrate binding domain (SBD) each represented. We show that BiP monomers can come together to form a high affinity (*K*_D_ of 9 nM) SBD‐linker dimer on the left of schematic or lower affinity (*K*_D_ of 108 μM) NBD–NBD dimer as shown on the right of the schematic. In the case of the high‐affinity (low *K*_D_) dimer on the left‐hand side: When ATP (in orange) or peptide substrate (represented as dark blue curved line) are introduced, the SBD binding interface is perturbed by allosteric (ATP) or steric (peptide) inhibition, resulting in the monomerization of BiP. Allosteric communication from ATP bound NBD to SBD is represented by gray arrow. When ADP (in green) reacts with this dimer form, no change is seen. In the case of the low‐affinity (high *K*_D_) dimer on the right‐hand side: Introduction of substrate, ATP, or ADP does not interfere with the binding interfaces in the NBD's and amounts of this dimer remain unaltered.

Considering our findings and existing structural data, BiP likely has two distinct protein‐binding interfaces: one in the SBD for unfolded or disordered chains, and another on the NBD surface for folded proteins (Figure 7). This dual‐interface model helps explain BiP's functional versatility in the ER.

In attempt to understand if the kinetics of either of the two dimer forms dominates *k*_on_ or *k*_off_ studies we used the equation relating molecular interaction kinetics and affinity:

$$K_{D}=\frac{k_{\mathrm{off}}}{k_{\mathrm{on}}}.$$

This produced a value of 3 μM (0.03/0.01), which is three orders of magnitude larger than the high‐affinity dimer *K*_D_ (0.009 μM), and two orders of magnitude smaller than the high‐affinity dimer *K*_D_ (108 μM). Considering this, we propose that in kinetics measurements, we are observing behavior of both dimer types, possible with some dominance of the high‐affinity (low *K*_D_) dimer. This makes sense as in the *k*_off_ studies, the majority of the dimer type being measured will be the low affinity (high *K*_D_) for as BiP starting concentration is 235 μM (both forms existing) but we dilute to and take measurements from 100 nM. This is 10 times the *K*_D_ value of the high‐affinity (low *K*_D_) dimer so most of these high‐affinity (low *K*_D_) dimers will remain intact, thus most monomerization observed will be contributed by the low affinity (high *K*_D_) form.

As well as the presence of mixed populations explaining this discrepancy between experimental and calculated kinetics and affinities, we can consider other mechanisms behind the fact that the parameters we measure here are more accurately described as *k*_obs_ as opposed to true *k*_on_.

Either one, or both, dimers may have more than one step in their binding modes. For example, as we see very slow assembly kinetics, this could reflect two steps, such as a weak encounter complex followed by a second rate reaction to form the final complex (Cornish‐Bowden, 1979; Hoare, 2021). Another possibility is the conformational gating effects of any of the BiP protomers (Weikl & Paul, 2014), particularly in the case of the high affinity (low *K*_D_), the SBD must be open to receive a linker of a BiP binding molecule, which in turn must be exposed. This is particularly worth considering in association studies as these involve monomerization by ATP and dimerization by ADP. We also acknowledge that the *k*_on_ measurements will reflect the dimerization in the presence of ADP. Although most studies suggest that BiP‐ADP has very similar characteristics to the apo state, these results could suggest some differences between the two, at least in the context of dimer formation.

### 4.2 BiP dimer formation as an intricate regulation mechanism

Dimerization (or higher‐order oligomer) formation is a very general control mechanism in biology. It allows the regulation of almost every biochemical process in our cells (Marianayagam et al., 2004). Then, being able to change the dimerization state (via ligand or protein concentration, etc.) is essential for its function. Our study shows how BiP's oligomerization is in turn regulated by different ligands. Our MP results show that adding both ATP and peptide does not further reduce dimer levels compared to ATP alone, suggesting that ATP saturates the monomerization response. The fact that PP28 does not influence BiP's ATPase activity (Figure S8) supports the idea that both ATP and peptide activate BiP by promoting monomerization, as previously proposed (Blond‐Elguindi, 1993). Structural studies suggest that linker engagement drives these changes and promotes the inactivation of BiP via dimerization (Preissler et al., 2015).

These mechanisms have important physiological implications. BiP activity must be tightly regulated given its roles in protein folding and ER quality control. Oligomerization provides a way to sequester BiP in an inactive form, with rising levels of unfolded peptides or ATP promoting monomer release and activation. However, increased BiP concentration and substrate availability may also trigger formation of low‐affinity dimers to re‐sequester excess active monomers, creating a feedback loop for homeostasis.

Although the exact ATP concentration in the ER is unknown, it is estimated to be in the low millimolar range, influenced by cytosolic ATP levels (1–5 mM) and ER‐specific transport mechanisms (Depaoli et al., 2019; Takaine et al., 2022; Vishnu et al., 2014; Yong et al., 2019). BiP itself will also influence ER ATP availability via its hydrolysis to ADP, reinforcing this model of concentration‐dependent regulation.

Homeostatic BiP levels in the ER have been experimentally derived as between 5 and 500 μM (Bakunts et al., 2017; Ghaemmaghami et al., 2003; Guth et al., 2004; Zimmermann & Lang, 2020), and to increase 12.5‐fold into the low millimolar range under stress (Bakunts et al., 2017). To apply our findings to physiologically relevant conditions, we used *K*_D_ values to model the prevalence of BiP monomer and dimer up to 5 mM in the presence and absence of 2 mM ATP, a near physiological concentration (Depaoli et al., 2019; Takaine et al., 2022; Vishnu et al., 2014; Yong et al., 2019) (Figure S9). Under physiological conditions, the prevailing dimer will be the one with the low affinity *K*_D_ and the other dimer will be disrupted by the high concentration of ATP reported inside the ER. For example, under high unfolded protein load, BiP dimerization is said to be in direct competition with the unfolded protein substrate (Blond‐Elguindi et al., 1993; Preissler et al., 2015; Preissler et al., 2020). Considering our results that suggest that BiP forms dimers via interactions between two NBD's of protomers, BiP dimerization could also compete with and depend on complex formation with other binding partners such as co‐chaperones and UPR transducers.

BiP has also been shown to oligomerize upon membrane association involving interactions with the NBD (Dores‐Silva et al., 2020). This mirrors BiP inactivation via transmembrane protein binding in the UPR (Alfaro‐Valdés et al., 2018). The NBD interactions driving membrane‐induced oligomerization align with our proposed low‐affinity dimer model, possibly reflecting a regulatory mechanism activated when BiP levels exceed the cellular demand for active chaperones.

Furthermore, the protein‐binding ability of the NBD in the ATP‐bound state serves as a regulatory mechanism of interaction with co‐chaperones including J‐domain proteins such as Hsp40 partners or the SEC61 translocon (Alder et al., 2005; Hamman et al., 1998).

Altogether, BiP exists in multiple forms—monomers, two dimer types, and potentially multiple forms of higher‐order oligomers—with distinct interaction surfaces and regulatory roles. Our findings support a model for BiP where oligomerization fine‐tunes its functional state to allow it to perform many roles. Considering this has high importance in further understanding its physiological function, for example, BiP AMPylation has also been shown to promote oligomerization (Perera et al., 2019); and in other HSP70s, AMPylation enhances disassembly of amyloid fibrils (Truttmann et al., 2018).

This work presents the first systematic single‐molecule analysis of BiP oligomerization, demonstrating the co‐existence of two distinct dimer forms, each previously resolved by crystallography (Preissler et al., 2020; Yang et al., 2017). We show that their formation is concentration‐dependent and functionally distinct, providing deeper insight into BiP's physiological regulation. While this study does not directly address higher‐order BiP oligomers, their prevalence at micromolar concentrations—relevant to physiological conditions—warrants further investigation (Gilchrist et al., 2006; Guth et al., 2004). MP and complementary techniques offer a promising path for future exploration.

## AUTHOR CONTRIBUTIONS

**Karina New**: Data curation (equal); formal analysis (equal); investigation (equal); methodology (equal); validation (equal); visualization (equal); supervision (equal); project administration (equal); writing—original draft (equal). **Miguel I. A. Lagos‐Espinoza**: Investigation (equal); methodology (equal); writing—original draft (equal). **Roi Asor:** Methodology (equal); writing—review and editing (equal). **Nathalie Casanova‐Morales**: Methodology (equal); supervision (equal); writing – review and editing (equal). **John William Young**: Investigation (equal); writing—review and editing (equal). **Zahra Alavi**: Investigation (equal); methodology (equal); supervision (equal); validation (equal); resources (equal); writing—original draft (equal). **Christian A. M. Wilson**: Conceptualization (equal); data curation (equal); funding acquisition (equal); investigation (equal); methodology (equal); project administration (equal); resources (equal); supervision (equal); validation (equal); writing—original draft (equal).

## FUNDING INFORMATION

This work was supported by Vicerrectoría de Investigación y Desarrollo (VID) of Universidad de Chile ENL 10/22 and Fondo Apoyo Pago de Publicaciones Científicas de la DI&DITT‐FaCiQyF, DI‐FaCiQyF, University of Chile, project number: DI‐FaCiQyF005 and Fondo Nacional de Desarrollo Científico y Tecnológico 1260037 (Christian A. M. Wilson). Fondo de Equipamiento Científico y Tecnológico, FONDEQUIP (EQ240012) to Karina New and Christian A. M. Wilson. John William Young was supported by a postdoctoral fellowship from the Canadian Institutes of Health Research (CIHR)—MFE181888. Roi Asor was supported by European Molecular Biology Organization long‐term postdoctoral fellowship (ALTF‐198‐2020) and is supported by the Engineering and Physical Sciences Research Council (EP/T03419X/1).

## CONFLICT OF INTEREST STATEMENT

All co‐authors have no competing interests.

## Supporting information


**Data S1.** Supporting Information.

## Data Availability

The data that supports the findings of this study are available in the main text and Supporting Information S1 of this article. Further information can be provided upon request.
