# Supplementary material for: Immunoglobulin binding protein (BiP) forms two types of dimers
Source: Protein Sci. 2026 Jul 9;35(8):e70690. doi: 10.1002/pro.70690 (PMC13347316; doi:10.1002/pro.70690)
Supplement: Supplementary file 1 — Data S1. Supporting Information. [file PRO-35-e70690-s001.docx]

**SUPPLEMENTARY MATERIAL**

**Immunoglobulin binding protein (BiP) forms two types of dimers**

Karina New^1^, Miguel I.A. Lagos-Espinoza^1^, Nathalie Casanova-Morales^2^, Roi Asor^3,4^, John William Young^3,4^, Zahra Alavi^5,^*, Christian A. M. Wilson^1,^*

^1^ Departamento de Bioquímica y Biología Molecular, Facultad de Ciencias Químicas y Farmacéuticas, Universidad de Chile, Chile

^2^ Facultad de Artes Liberales, Universidad Adolfo Ibáñez, Santiago, Chile

^3^ Physical and Theoretical Chemistry Laboratory, Department of Chemistry, University of Oxford, Oxford, UK

^4^The Kavli Institute for Nanoscience Discovery, Oxford, UK

^5^ Department of Physics, Loyola Marymount University, Los Angeles, CA, USA

* Corresponding authors: ZA, [zahra.alavi@lmu.edu](mailto:zahra.alavi@lmu.edu); CAMW, [yitowilson@gmail.com](mailto:yitowilson@gmail.com)

Correspondence should be addressed to:

**Christian A.M. Wilson,** Departamento de Bioquímica y Biología Molecular, Facultad de Ciencias Químicas y Farmacéuticas, Universidad de Chile, Chile. Email: [yitowilson@gmail.com](mailto:yitowilson@gmail.com). Tel: +56-2-29771904. https://orcid.org/0000-0002-6499-6957

**Zahra Alavi,** Department of Physics, Loyola Marymount University, Los Angeles, CA, USA. Email: [zahra.Alavi@lmu.edu](mailto:zahra.Alavi@lmu.edu). Tel: +13102582399. https://orcid.org/0000-0001-9760-174X


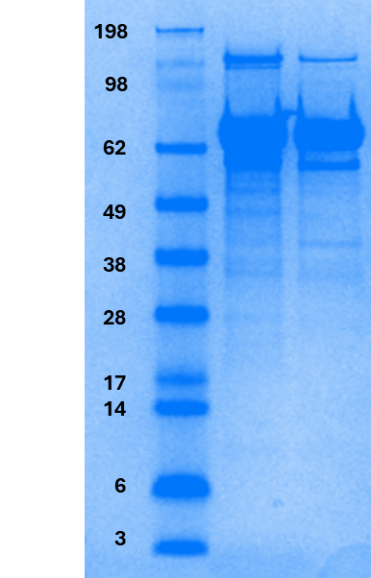
BiP purity assessment

**Figure S1 – Purified BiP.** LDS-page gel of BiP purification (performed as outlined in main text materials and methods and Ramírez et al., 2017 and Rivera et. al., 2023) stained with Comassie Blue. Lane 1: Invitrogen Ladder 26630, lane 2: 20 µg BiP in solution, lane 3: 4 µg BiP in solution.

Verification of good BiP adhesion to glass surface in MP

It is well outlined in literature that although mass photometry is detecting events on the glass surface, measurements reflect the molecular state in solution (Young et. al., 2018; Kratchovil et. al., 2024). The fact that the recording of the landing event is not discriminated by landing orientation also supports this and the suitability of this approach to measure distinct conformations of the same stoichiometry. We confirmed the lack of surface binding artifacts when using the system to measure BiP species by observing the very low level of molecule unbinding events from glass surface (Figure S2). Unbinding events are seen as positive contrast values that convert to a negative mass value [1, 2] and correlate to poor binding of the protein with the glass surface and thus improper detection and measurement. This is not the case in these studies. Figure S2 clearly shows that measurement of BiP from low (10 nM) and high (235 µM) incubation concentrations show very negligible unbinding events (0.3 and 0.7 %, respectively).


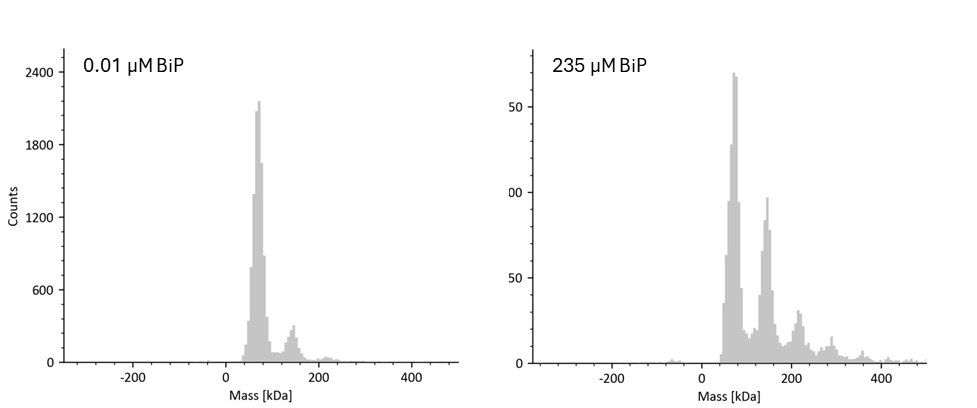


**Figure S2 – Negligable unbinding of BiP from glass surface in mass photometry.** Representative histograms of low and high BiP incubation concentrations show extremely low amount of molecular unbinding from the measurement slide (0.3 and 0.7 %, respectively). This demonstrates that in mass photometry, BiP is detected and measured in a very robust manner.

Variation in the detection of oligomeric state of BiP during data acquisition

To rule out possible dimer detection bias effects, we examined the 60 second video data across 3 divisions of 20 seconds each. This showed that D:M value does not vary significantly between each 20 seconds within one video, and this is seen at high, medium, and low concentrations (Figure S3). This also demonstrates that rapid dilution from incubation concentrations (10 nM – 230 µM) to measuring concentration (10 nM) does not cause any dissociation of dimers at time of measurement as monomer and dimer distributions remain constant.


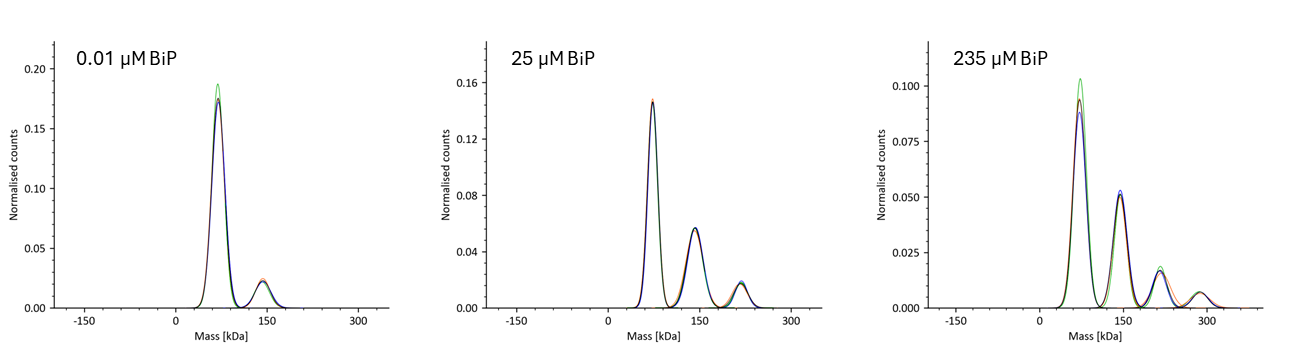


***Figure S***3 **-** **Negligible variation in the detection of oligomeric state of BiP during data acquisition.** MP mass histograms of rapidly diluted BiP protein after applying the dilution protocol (see Methods section). Each movie was divided into three 20 s intervals and histograms of the landing events during each interval were plotted (blue, orange and green lines, sequentially) and shown here together alongside the histogram generated by analyzing the full video (black line).

Mass Photometry Derivation of BiP Oligomer Species Concentration and Formation Patterns

The MP data was used to calculate the concentration of each BiP species within the reactions by the equation:

$$\left[ Spec \right]= {Count}_{Spec}{* M}_{Spec}* \frac{M+2D+3Tr+4Te}{[TBiP]}$$

Whereby [Spec] is the concentration of the species being calculated, M_Spec_ is the number of monomeric subunits within the species (i.e., 1 for monomer, 2 for dimer etc), Count_spec_ is the absolute count of the species being calculated, [TBiP] is the total experimental concentration of BiP used in the reaction, M the total monomer count, D the total dimer count, Tr the total trimer count and Te the total tetramer count.

From these values, K_D_ values of dimer formation are calculated using the equation:

$$K_{D}=\frac{[{M]}^{2}}{[D]}$$

As it was observed that these values differed with total concentration of BiP, (Figure S4), we questioned if this was due to the formation of more than one dimer structure, or an effect of the formation of higher order oligomers. However, as a sequential decrease in dimer, trimer, and tetramer prevalence is observed, we consider the differences in K_D_ values due to differences in the predominant dimer conformation present at different concentrations.

As studies into D:M as a function of total BiP concentration showed a biphasic relationship, with two possible K_D_ regimes of dimer formation, we wanted to confirm this possibility by using another approach to obtain K_D_ values and eliminate the possibility of higher order species formation having an impact on this.

K_D_ values were calculated from concentration values derived from MP data as outlined in the main text. When these values are plotted as a function of total BiP concentration, we observe a gradual increase of K_D_ value before these values stabilize at a plateau around 55 µM after the BiP total concentration reaches 15 µM (Figure S4A). The fact that we observe a concentration dependent shift in K_D_ values supports the idea that two distinct dimer constructs are being formed by BiP, or that the formation of the higher order species is influencing the ability of dimers to assemble.

To investigate higher order oligomer formation pattern, the ratio of dimer to monomer was compared to the ratios of all species (monomer, dimer, tetramer and trimer) to monomer and all oligomer species (dimer, trimer and tetramer) to monomer. As all of these values exhibit equal patterns when plotted as a function of total BiP concentration (Figure S4B), we can infer that formation of high order oligomers such as trimers and tetramers do not sequester monomeric units available to create the dimers. Thus, oligomers are formed by sequential addition of monomer to each construct, (i.e., monomer – dimer interaction to form a trimer, monomer – trimer interaction to form a tetramer etc.) as opposed to their direct formation from monomeric subunits, for example by three monomers directly interacting to form the trimer etc. This model of oligomer assembly supports the idea that the discrete K_D_ value classes are a result of distinct dimer structures.

A B

**Figure S4 – Dimerisation shows two concentration dependent classes that is not a result of higher order oligomer effects. (A)** K_D_ values of BiP dimerisation from different total concentrations of BiP are calculated from monmer and dimer concentrations in soulutuoin as calculated from total counts of monomer and dimer species from MP studies. **(B)** Fractions of BiP oligomer species in relation to monomer presence, ploted as a functionof total BiP concentration.

Statistical error and reliability of oligomer quantification

In order to verify the statistical error and reliability of oligomer quantification, logarithmic scale values of KDE’s were plotted as a function of mass (Figure S3). From this, it is seen that expected mass peaks corresponding to BiP monomer, dimer, trimer and tetramer values correlate well to their expected masses across the concentration range used. The linear decrease in probability of mass measurement (i.e., no dominance of any oligomer peak) shows that there is no bias towards the detection of any BiP species in MP. Around 300 kDa, mean values begin to appear unstable and error margins increase, particularly at low concentrations. As such, oligomers larger than tetramers are not considered in this work. This outcome also serves to support the idea that BiP oligomers are formed by sequential addition of monomer to each construct as outlined above.


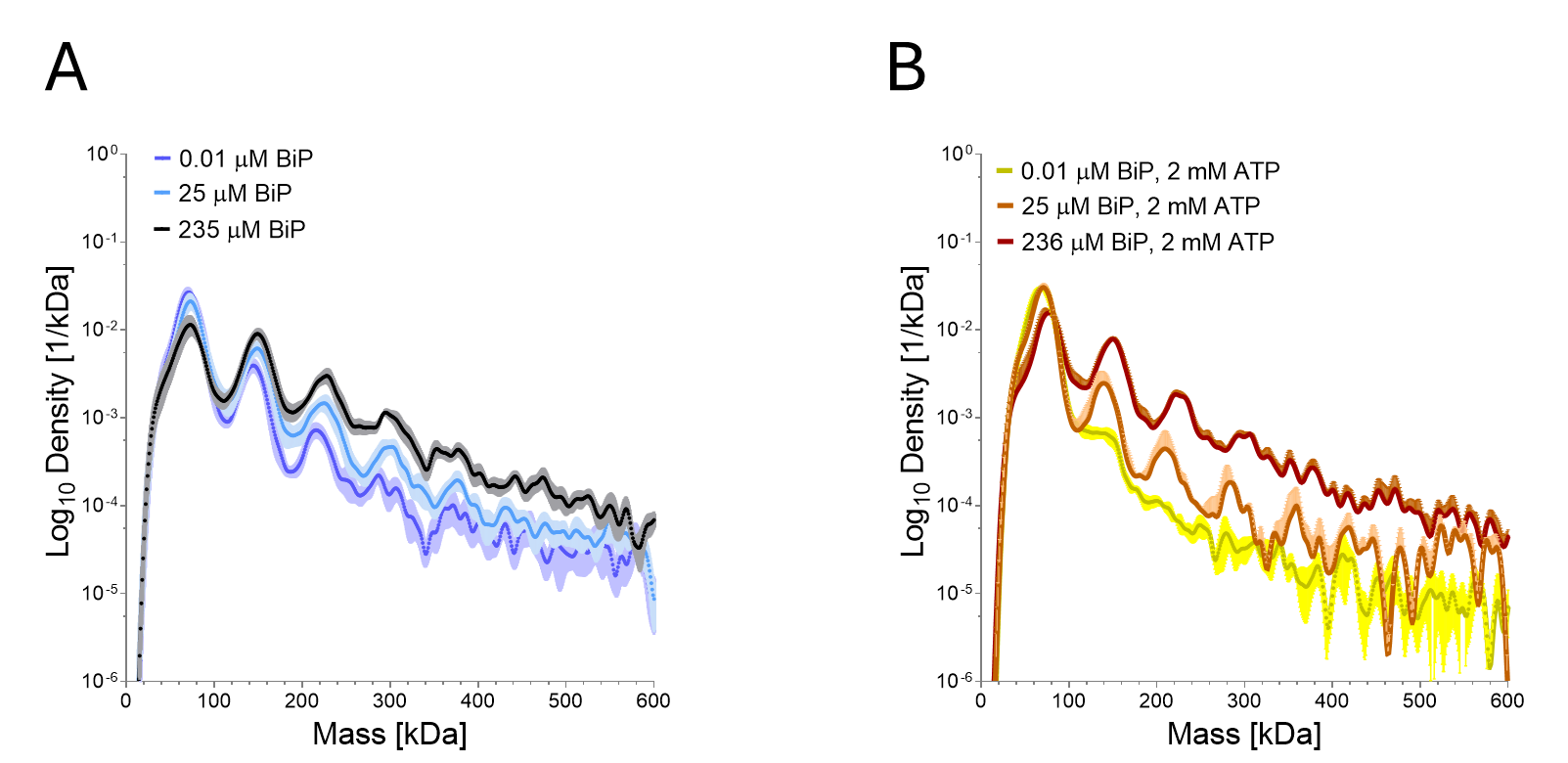


**Figure S5- Logarithmic scale of KDE values**. All data for each concentration are plotted and displayed in the absence **(A)** and presence **(B)** of ATP to observe reliability of mass photometry measurements of BiP oligiomer formation. Solid lines are average values and shadows standard error values.

Akaike Information Criterion Analysis

To statistically verify which of the monophasic or biphasic hyperbole equation fit best to the data of BiP dimerization in the absence of ATP, and the difference in Akaike Information Criterion value (Akaike 1974) was calculated as 96.74 with the preferred model being the biphasic equation, with correctness probability of >99%. This very strongly confirms that the BiP dimerization relative to total concentration is biphasic, with two K_D_ values, and thus two distinct BiP dimers exist. This was not necessary in the case of dimerization in the presence of ATP as the statistical software (Graphad 8) was unable to fit the biphasic curve to this data

BiP individual molecule counts during dissociation

To confirm no measurement bias towards any BiP species, the k_off_ data was scrutinized by quantifying the total number of individual BiP molecules counted at each measured time point by:

Count_tx_ = M + 2D + 3Tr + 4Te

whereby Count_tx_ represents the total number of individual BiP molecules at given time point (tx), M is the total monomer count, D the total dimer count, Tr the total trimer count and Te the total tetramer count. This revealed only small changes in Count_tx_ values that were not dependent on time. The mean and standard deviation values of Count_tx_ of all time points for 5 individual experiments are shown in table S1.

| **Exp n.** | **T0** | **T1** | **T2** | **T3** | **T4** | **T5** | **MEAN** | **SD** |
| --- | --- | --- | --- | --- | --- | --- | --- | --- |
| 1 | 4057 | 3239 | 3375 | 3319 | 2293 | N/A | 3257 | 563 |
| 4 | 5957 | 5304 | 6404 | 6439 | 5484 | 4594 | 5697 | 650 |

***Table S1* - Values of total monomer molecule count at different time points for 5 individual experiments.** For each experimental dataset, total BiP molecule values were calculated for each time point measurement by M + 2D + 3Tr + 4Te, whereby M is the total monomer count, D the total dimer count, Tr the total trimer count and Te the total tetramer count. Here, count values and mean and standard deviation are shown for 2 experiments

To be able to compare this information across datasets with intrinsic differences in total molecular counts (due to factors such as pipetting differences), we performed normalization by dividing each Count_tx_ value by the median value of that dataset:

Count_tx_(norm) = Count_tx_ / (Med)Count_t1_,Count_t5_

These and their mean and S.D. values are reported in table S2.

| **Exp n.** | **T0** | **T1** | **T2** | **T3** | **T4** | **T5** | **MEAN** | **SD** |
| --- | --- | --- | --- | --- | --- | --- | --- | --- |
| 1 | 1.22 | 0.98 | 1.02 | 1.00 | 0.69 | N/A | 0.98 | 0.17 |
| 2 | 0.99 | 1.39 | 0.90 | 1.00 | 1.19 | N/A | 1.09 | 0.18 |
| 3 | 1.19 | 0.90 | 1.10 | 1.00 | 0.96 | N/A | 1.03 | 0.10 |
| 4 | 1.04 | 0.93 | 1.12 | 1.13 | 0.96 | 0.80 | 1.00 | 0.11 |
| 5 | 0.84 | 1.15 | 1.34 | 0.60 | 1.14 | 0.93 | 1.00 | 0.24 |

Table S2 – Normalised Count_tx_ values of each time point measurement of 5 individual experiments. Mean and standard deviation values are also reported for each dataset.

The total individual BiP molecule count across all species is constant at different times, regardless of the fact that the distribution of oligomer species changes, showing there is no bias towards detection of a particular oligomer species.

Untreated data of BiP dimer association kinetics

To study k_on_ values of BiP dimerization, 2 mM ATP was added to 120 µM BiP and MP measurements taken immediately and at time intervals until a plateau reached. The D:M values of these measurements were then plotted against time and a one phase association curve fitted (Figure S5). From this, k_on_ of 0.08 µM^-1^ min^-1^ and tau of 129.8 min were obtained. To achieve true k_on_ values, association must be measured from the point at which the ATP added to the reaction to monomerize BiP was fully converted to ADP (that will stabilize dimers). From ATPase activity assays, this was calculated to be 60 minutes (main text Figure 2C). From original plots of BiP D:M vs time in these experiments (Figure S5), the D:M at 60 minutes was seen to be 0.28. Thus, 60 was subtracted from all time point values and 0.28 subtracted from all D:M values to obtain true time = 0 min and D:M = 0 points. These new data are plotted and a one phase association curve fitted (main text Figure 2D).

**Figure S6 – Untreated data of time vs BiP D:M.** 2 mM ATP is added to 120 µM BiP and mass photometry measurements taken immediately and at time points until D:M plateau reached. The one-phase association curve fitting was then used to identify D:M value at 60 minutes is 0.28 to treat all data to obtain true BiP dimer association kinetics by subtracting 60 and 0.28 from time point and D:M values, respectively (main text Figure 2D).

Nano‐rheology experimental setup

To study mechanical properties of BiP upon ligand binding, we employed nano‐rheology experimental setup (Figure S6). BiP was directly tethered between a gold film surface evaporated on a glass slide and 20 nm diameter gold nano-particules (GNPs) (Cytodiagnostics, USA), constituting the lower part of a thick flow chamber. Gold coated slides and coverslips are prepared by evaporating 3 nm Cr followed by 30 nm Au on glass slides and coverslips using an e‐beam evaporator machine (CHA MARK 40). Preparing the flow chamber with BiP on it takes 3 days. BiP was attached to both gold surfaces through two exposed cys residues inserted by site‐directed mutagenesis, at positions 185 and 537, located in the NBD and SBD, respectively (Casanova-Morales et al., 2018)

On the first day, BiP protein is first diluted to a final concentration of 2 μM in an experimental buffer, containing KCl 200 mM, HEPES 20 mM, MgCl_2_, EDTA 100 μM, pH 7. This is the optimum pH which minimizes the nonspecific binding of BiP to gold. Then 500 μL of the protein solution is inserted into a bordered area in the slide for overnight at room temperature.

On the second day the slide is first rinsed with distilled water in order to remove the unbound proteins and the imidazole from the solution where BiP was kept after purification. The slide is then rinsed once again and immersed in 20 nm GNPs for ∼90 min. When GNPs were bound the slide turns slightly red. To negatively charge the GNPs, they were immersed in KH_2_PO_4_ 1 M pH 4 and incubated with 2 μM thiol‐modified single stranded (ss) DNA (/5ThioMC6‐D/AAAAAAAAAAAAAAAAAAAACGCATTCAGGAT), overnight. Thiol‐modification enables the DNA to bind to gold because of the S‐H group.

On the third day, first the unbound DNA is washed away with distilled water and then the slide is immersed in 500 μL in the experimental buffer previously mentioned. Finally, to close the chamber, a gold‐coated cover slip was arranged at 200 µm distance, constituting the upper part of the chamber, closing it, and acting like a capacitor arrangement. After this a fresh chamber that last 8 h is ready to be used(Casanova-Morales et al., 2018).

*Nano‐rheology experimental measurements*

Only chambers prepared in the way previously mentioned, until the third day, must be used for nano‐rheology experiments. Applying an alternating current (AC) potential difference between the two gold films, generated a mechanical response in the protein, measured as an averaged deformation (Amplitude)(Alavi et al., 2015). Using this experimental setup, two types of determinations were performed: BiP mechanical response to an applied force in absence and presence of ATP, and PP28 dissociation constants (*K*_D_) determinations (Blond-Elguindi et al., 1993). All the nano‐rheogical determinations were performed using the experimental buffer, working at room temperature.

*BiP mechanical response to an applied force*

In these experiments, we studied BiP viscoelastic behavior applying a force over the protein, using increasing concentrations of pp28 (BioServ, UK) substrate peptide in absence and presence of ATP. To apply the force, the frequency used was 12 Hz, fixing the amplitude of the applied voltage (0.3 mV). The ligand concentrations used were 2 mM ATP (Sigma) and pp28 peptide substrate from 0 to 100 μM. Each curve in the graph corresponds to the average of five experimental runs.


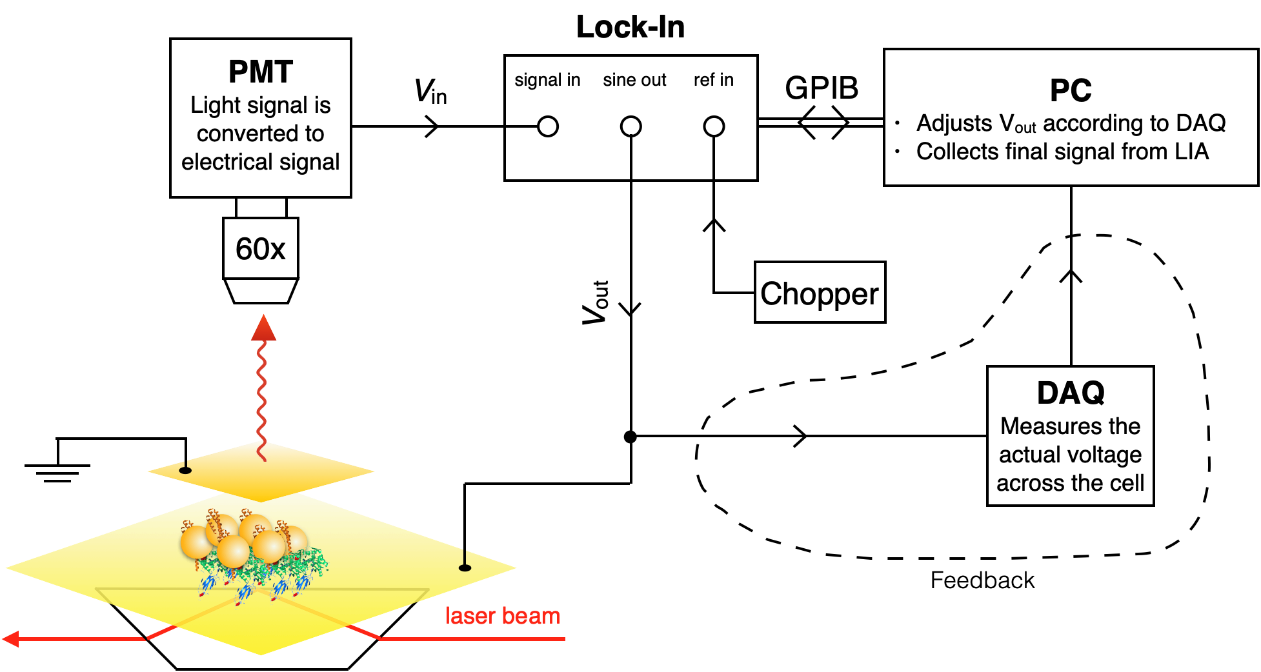
To describe BiP mechanical response, the averaged deformation of the protein subjected to the oscillatory force was measured and compared to the Maxwell model of viscoelasticity(Alavi et al., 2015):

$$|z|=F0/\gamma\omega\sqrt{1+{(\omega/\omega c)}^{2}}$$

**Figure S7 -** **BiP nano-rheology experimental setup.** This image shows the flow chamber where BiP is attached to both gold-coated surfaces, where they work as parallel plates capacitor for mechanical excitation, and the evanescent wave scattering optics is used as a signal. BiP was bound between a gold film surface evaporated on a glass slide and 20 nm diameter GNPs, which corresponds to the lower part of the flow chamber. To close the circuit, a gold-coated cover slip was arranged at 200 μm distance, which corresponds to the upper part of the flow chamber, acting like a capacitor arrangement. Two exposed cys residues at position 185 and 537, located in the NBD and SBD respectively, are used to attach BiP to the GNP. An oscillatory force is applied over BiP by placing the protein between two conducting plates and run a voltage across them. GNPs are covered with ssDNAs on the surface to negatively charge them.

In the context of our experiments, the corresponding deformation amplitude is |*z*|, frequency is ω, ω_C_ parameter is κ/γ where κ is an elastic parameter (dimensions of force/length) and γ is a dissipation parameter (dimensions of mass/time). Finally, *F*_0_ is the amplitude of the applied force. In our case in the graphs ω and |z| correspond to frequency and amplitude axis, respectively, and then the data was fitted using the following expression:

$$Amplitude=A/Frequency\sqrt{1+ {(Frequency/B)}^{2}}$$

where *A* = *F*_0_/γ, then the dissipation parameter γ is proportional to 1/*A* and *B* = ω_c_ = κ/γ ∝ κ*A*, then the elastic parameter κ is proportional to *B*/*A*

*Peptide dissociation constant (K_D_) determination*

With our experimental setup, the other assay performed was *K*_D_ determination for BiP peptide ligand. The peptide pp28 *K*_D_ determinations were performed both in absence and presence of the ATP. To achieve this, force was maintained in a constant frequency of 12 Hz, 2 mM ATP and pp28 concentration was varied in a range between 0 and 100 μM. Each point in the graph corresponds to the average of five measurements. For the binding isotherm measurements, the following expression was considered:

|z|=α+K_D_/[pp28]

where [pp28] is the pp28 concentration used, *K*_D_ is a dissociation constant determined, and α is a normalization factor.

| **Technique** | **Ligand** | **K_D_** (µM) | **Reference** |
| --- | --- | --- | --- |
| *Equilibrium dialysis* | ATP | 0.2 ± 0.02 | (Hendershot et al., 1995) |
| *Nanorheology* | ATP | 0.96 ± 0.21 | (Casanova-Morales et al., 2018) |
| *Mass photometry* | ATP | 0.13 ± 0.05 | Current study |
| *Nanorheology* | PP28 | 1.54 ± 0.75 | Current study |
| *Mass photometry* | PP28 | 1.0 ± 0.04 | Current study |

***Table S3 –* Dissasociation constants of BiP and ligands as determined by different approaches.** Here we present mass photometry data that allows determination of protein-ligand binding parameters via monitoring changes in dimerization. Concordance with previously determined values supports the validity of this approach.

BiP Enzymatic Activity in peptide presence

ATPase activity of BiP was performed as described in the main text with the addition of varying concentrations of PP28 from 0 to 50 µM. ATP concentration data were then plotted against respective time in seconds in Graphpad Prism 8.0 and linear regression analysis performed to obtain slope values. Slope values for each concentration of PP28 used were then normalized via their division by the slope of ATP activity with no peptide present. T-tests performed on these normalized values and statistics described.


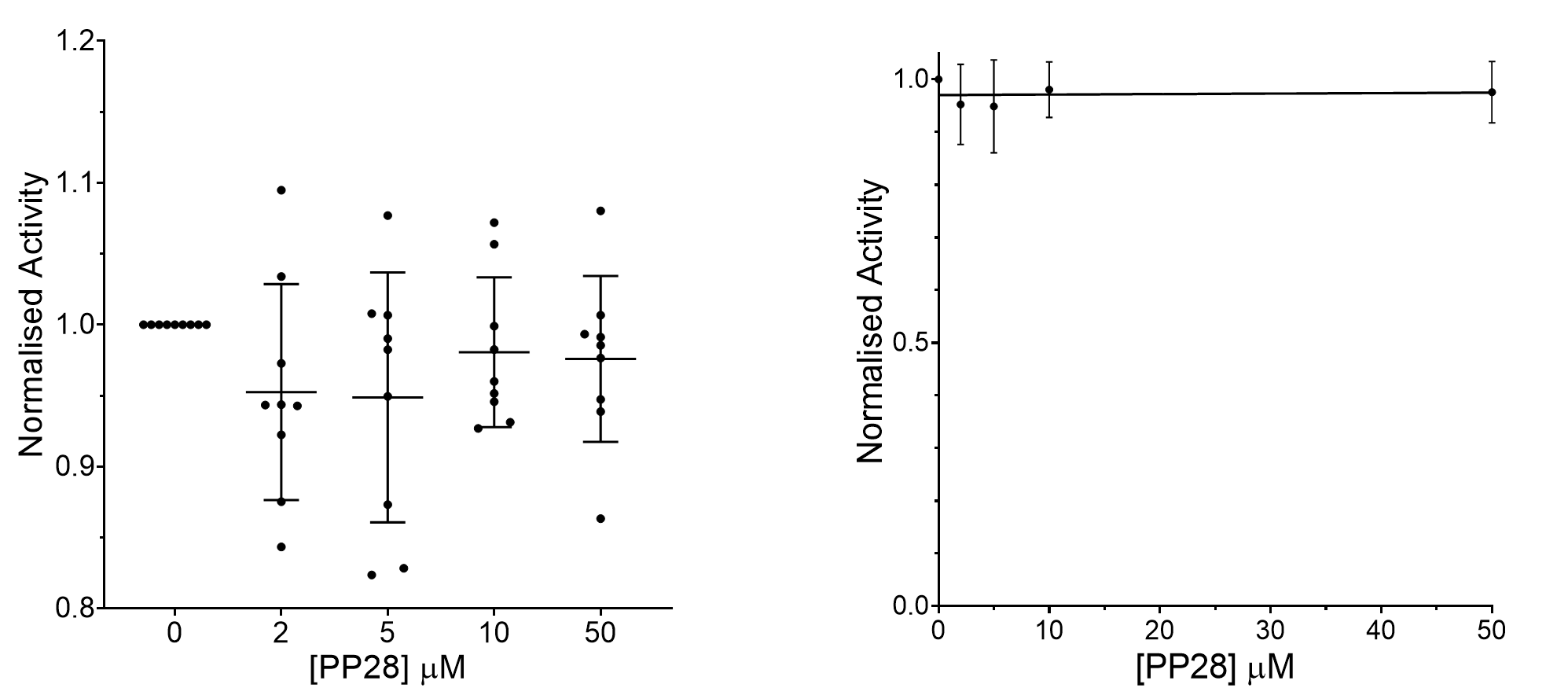
A B

**Figure S8 – ATPase activity of BiP in the presence of varying concentrations of peptide. (A)** Individual points of slope values of ATPase activity of BiP calculated over time. All points are normalised to the slope value of reactions containing 0 µM PP28 taken on the same day. **(B)** Linear regression of the normalised slope values plotted as a function of PP28 concentration.

*Results*

To investigate the allosteric activity of BiP in regard to PP28 interaction, assays of BiP ATPase capacity as a function of PP28 concentration were performed. Here, spectrophotometry is used to quantify presence of an enzymatically linked reaction product of ATP hydrolysis to ADP to ascertain changes in ATP binding and/or hydrolysis. Interestingly, no changes in this behavior were observed with addition of up to 50 µM of PP28. Over the course of 1 hour, ATP levels were measured to have fallen to around 30 % of those at time point 0 in the absence of PP28 as well as with increasing concentrations of the peptide (Figure S6). The rates of this reaction at varying concentrations of PP28 were calculated (2.19 x 10^-4^ ± 2.75 x 10^-5^ and 2.14 x 10^-4^ ± 2.64 x 10^-5^ 1/s with 0 µM and 50 µM of PP28, respectively) and normalized to the rates observed at 0 µM PP28. These values are then plotted against the concentration value. Normalized values are all around 1, revealing that BiP ATPase activity measured here does not significantly differ across the range of PP28 concentration employed, indicating that this behavior is not influenced in any way by PP28 occupancy of SBD.


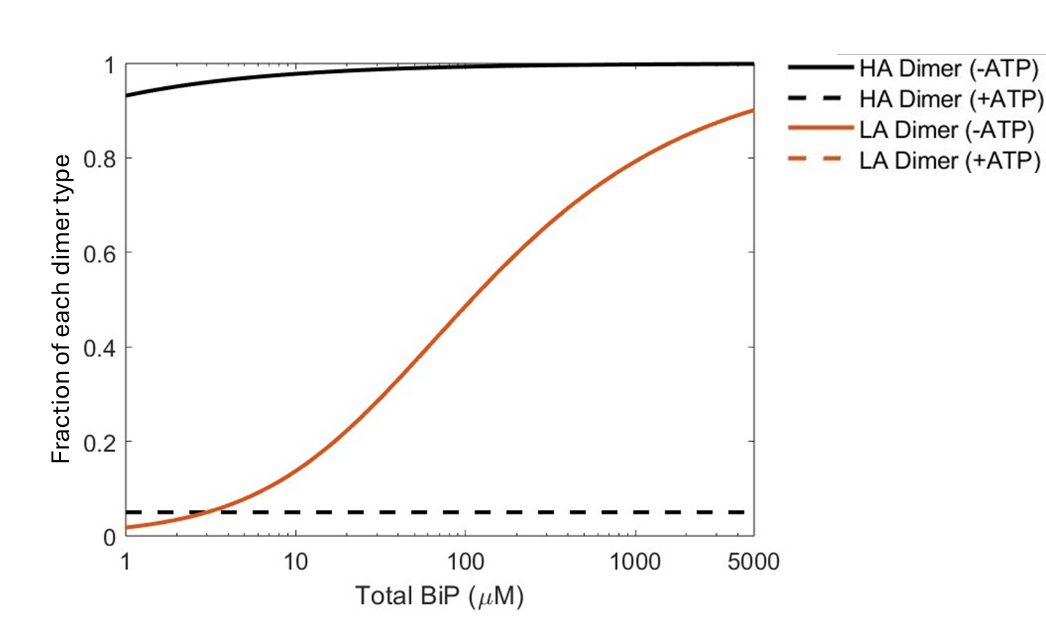


Figure S9 - Schematic showing fraction of total protein occupied by each dimer type in the presence and absence of ATP at physiological concentrations of BiP in the ER. With no ATP, the high affinity (HA) dimer would be extremely high due to its low K_D_ value (solid black line). However, as there is a high concentration of ATP in the ER lumen, and this dimer type is extremely sensitive to disruption by ATP, the true prevalence would be kept very low (dashed black line). The low affinity (LA) dimer would slowly increase with concentration due to its relatively high K_D_ (solid orange line). This dimer is resistant to ATP effects so shows no change in behavior in the presence of ATP (dashed orange line).

References

Akaike, H. (1998). A New Look at the Statistical Model Identification. In E. Parzen, K. Tanabe, & G. Kitagawa (Eds.), *Selected Papers of Hirotugu Akaike* (pp. 215–222). Springer New York. https://doi.org/10.1007/978-1-4612-1694-0_16

Alavi, Z., Ariyaratne, A., & Zocchi, G. (2015). Nano-rheology measurements reveal that the hydration layer of enzymes partially controls conformational dynamics. *Appl. Phys. Lett.*, *106*(20), 203702. https://doi.org/10.1063/1.4921414

Blond-Elguindi, S., Cwirla, S. E., Dower, W. J., Lipshutz, R. J., Sprang, S. R., Sambrook, J. F., & Gething, M. J. H. (1993). Affinity panning of a library of peptides displayed on bacteriophages reveals the binding specificity of BiP. *Cell*, *75*(4), 717–728. https://doi.org/10.1016/0092-8674(93)90492-9

Casanova-Morales, N., Quiroga-Roger, D., Alfaro-Valdés, H. M., Alavi, Z., Lagos-Espinoza, M. I. A., Zocchi, G., & Wilson, C. A. M. (2018). Mechanical properties of BiP protein determined by nano-rheology. *Protein Science*, *27*(8), 1418–1426. https://doi.org/10.1002/pro.3432

Hendershot, L. M., Wei, J. Y., Gaut, J. R., Lawson, B., Freiden, P. J., & Murti, K. G. (1995). In vivo expression of mammalian BiP ATPase mutants causes disruption of the endoplasmic reticulum. *Molecular Biology of the Cell*, *6*(3), 283–296. https://doi.org/10.1091/mbc.6.3.283

Kratochvíl, J., van Wee, R., Thiele, J. C., Loewenthal, D., Bardzil, J., Iqbal, K., ... & Kukura, P. (2025). Best practice mass photometry: a guide to optimal single-molecule mass measurement. *Nature Protocols*, 1-25.

Young, G., Hundt, N., Cole, D., Fineberg, A., Andrecka, J., Tyler, A., ... & Kukura, P. (2018). Quantitative mass imaging of single biological macromolecules. *Science*, *360*(6387), 423-427
